# Bioenergetic modulation with dichloroacetate reduces the growth of melanoma cells and potentiates their response to BRAF^V600E^ inhibition

**DOI:** 10.1186/s12967-014-0247-5

**Published:** 2014-09-03

**Authors:** Cecilie Abildgaard, Christina Dahl, Astrid L Basse, Tao Ma, Per Guldberg

**Affiliations:** Danish Cancer Society Research Center, Copenhagen, Denmark; Department of Biology, University of Copenhagen, Copenhagen, Denmark

**Keywords:** Dichloroacetate, Melanoma, BRAF, Bioenergetics, Metabolism, ATP

## Abstract

**Background:**

Advances in melanoma treatment through targeted inhibition of oncogenic BRAF are limited owing to the development of acquired resistance. The involvement of BRAF^V600E^ in metabolic reprogramming of melanoma cells provides a rationale for co-targeting metabolism as a therapeutic approach.

**Methods:**

We examined the effects of dichloroacetate (DCA), an inhibitor of pyruvate dehydrogenase kinase, on the growth and metabolic activity of human melanoma cell lines. The combined effect of DCA and the BRAF inhibitor vemurafenib was investigated in BRAF^V600E^ -mutated melanoma cell lines. Vemurafenib-resistant cell lines were established *in vitro* and their sensitivity to DCA was tested.

**Results:**

DCA induced a reduction in glycolytic activity and intracellular ATP levels, and inhibited cellular growth. Co-treatment of BRAF^V600E^-mutant melanoma cells with DCA and vemurafenib induced a greater reduction in intracellular ATP levels and cellular growth than either compound alone. In addition, melanoma cells with *in vitro* acquired resistance to vemurafenib retained their sensitivity to DCA.

**Conclusions:**

These results suggest that DCA potentiates the effect of vemurafenib through a cooperative attenuation of energy production. Furthermore, the demonstration of retained sensitivity to DCA in melanoma cells with acquired resistance to vemurafenib could have implications for melanoma treatment.

**Electronic supplementary material:**

The online version of this article (doi:10.1186/s12967-014-0247-5) contains supplementary material, which is available to authorized users.

## Background

A hallmark of cancer is the reprogramming of cellular metabolism towards aerobic glycolysis. This metabolic pattern is characterized by increased glucose uptake and highly up-regulated glycolytic activity with fermentation of glucose into lactic acid instead of complete aerobic decomposition in the mitochondria. Aerobic glycolysis, also referred to as the Warburg effect, resembles the anaerobic metabolism of normal cells, but occurs in the context of an adequate oxygen supply [1]. The reprogramming of metabolism in cancer cells is a highly complex and heterogeneous process, which is driven by a wide variety of genetic and non-genetic strategies to overcome energy restriction [2–4].

The *BRAF*^*V600E*^ oncogene, present in more than 50% of melanomas [5], has been directly implicated in the reprogramming of cellular metabolism. The constitutive activity of mutant BRAF reduces the expression of oxidative enzymes and the number of mitochondria, while increasing the expression of glycolytic enzymes and lactic acid production [6,7]. Furthermore, a molecular link was recognized between the RAS-RAF-MEK-ERK-MAPK pathway and the energetic-stress check-point mediated by the liver kinase B1 (LKB1)-AMP activated protein kinase (AMPK) pathway, suggesting a role of BRAF^V600E^ in mediating resistance to energetic stress [8,9]. BRAF affects oxidative metabolism through microphthalmia-associated transcription factor (MITF)-dependent control of the mitochondrial master regulator PGC1α [7]. Previous studies have shown that melanomas expressing PGC1α have a more oxidative phenotype than PGC1α-negative melanomas [4,7]. In addition, BRAF^V600E^ was shown to mediate oncogene-induced senescence through metabolic regulation. This mechanism involves an increase in pyruvate dehydrogenase (PDH) activity through the suppression of pyruvate dehydrogenase kinase (PDK) [10]. PDH controls the coupling between glycolysis and mitochondrial respiration by facilitating the influx of pyruvate into the mitochondria, promoting complete utilization of glucose. The PDK-PDH axis is often dysregulated in cancer, where PDK over-expression reduces the coupling between the two energy systems and thereby contributes to the Warburg effect [11,12]. On the basis of these findings, targeted inhibition of PDK was proposed as a therapeutic option for melanoma, with a possible synergistic effect of chemical BRAF^V600E^ inhibitors, such as vemurafenib [10,13].

Dichloroacetate (DCA) is an inhibitor of the four isoforms of PDK and was previously used for treatment of lactic acidosis [14,15], with low toxicity at effective dose levels [16,17]. Several studies have demonstrated that DCA reverses the Warburg effect in cancer cells and negatively affects their growth and survival [13,18–21]. This effect was attributed to a normalization of the mitochondrial membrane potential from the hyperpolarized state that characterizes cancer cells. The changes in membrane potential result in the reopening of voltage-gated anion channels and were shown to introduce a re-sensitization to apoptosis, due to a regained ability to release pro-apoptotic mediators [18]. Here we have investigated the effect of DCA on melanoma cells. Specifically, we analyzed cellular responses with regards to metabolism, bioenergetics, growth, proliferation and cell death in melanoma cell lines, primary human melanocytes, and BRAF^V600E^-mutant melanoma cells with acquired resistance to vemurafenib.

## Methods

### Chemical compounds

DCA (sodium dichloroacetate) and 2-Deoxy-D-glucose (2-DG) were purchased from Sigma-Aldrich and dissolved in dH_2_O to working stock concentrations of 1 M. Vemurafenib (PLX4032) was purchased from Selleck Chemicals and dissolved in DMSO to a working stock concentration of 0.05 M.

### Cell culture

The melanoma cell lines ED-007, ED-013, ED-024, ED-027, ED-029, ED-034, ED-050, ED-070, ED-071, ED-117, ED-140, ED-179 and ED-196 were obtained from the European Searchable Tumour line Database (ESTDAB, ED) [22]. The melanoma cell line SK-MEL-28 was purchased from ATCC. Primary human epidermal melanocytes (neonatal) from lightly pigmented tissue (HEMn-LP) were purchased from Invitrogen. The melanoma cell lines were cultured at 37°C under 5% CO_2_ in RPMI-1640 medium supplemented with 10% fetal bovine serum and 1% penicillin/streptomycin. HEMn-LP cells were cultured under the same conditions in 254CF medium supplemented with 1% human melanocyte growth supplement (HMGS-2) and 12-*O*-tetradecanoyl-phorbol-13-acetate (TPA; 10 ng/ml). All media and supplements were purchased from Invitrogen.

### Metabolic analysis

Metabolic characterization was performed on melanoma cell lines and primary human melanocytes using a Seahorse XF96 extracellular flux analyzer (Seahorse Bioscience, Billerica, MA), which performs real-time measurements of extracellular acidification rate (ECAR) and oxygen consumption rate (OCR). An assay was designed to study the capacity of the mitochondrial and glycolytic energy systems. The ECAR and OCR were measured under basal conditions and during successive addition of five metabolic modulators: The ATP synthase inhibitor, oligomycin (1 μM); the mitochondrial membrane permeabilizer, carbonyl cyanide-*4*-(trifluoromethoxy)phenylhydrazone (FCCP) (1 μM); the inhibitors of mitochondrial respiration, rotenone (1 μM) and antimycin A (1 μM); and the glycolytic inhibitor, 2-DG (100 mM). The XF Cell Mito Stress Kit, containing oligomycin, FCCP, rotenone and antimycin A, was purchased from Seahorse Bioscience.

### ATP measurements

Intracellular ATP levels were measured using the ATPlite, 1 step Luminescence Assay System (Perkin Elmer), a method based on the reaction of ATP with luciferase and D-luciferin. The cells were seeded in triplicates with 10,000 cells per well and treated with the indicated compounds and vehicle control for 2 or 24 hours. Luminescence was measured with Spectra Max Gemini EM luminescence microplate reader (Molecular Devices) and normalized to background levels.

### Crystal violet assay

A crystal violet assay was applied to evaluate the effect of the studied compounds on cell growth. Cells were seeded in duplicates at a suitable density and then treated with DCA, vemurafenib, the two compounds combined and vehicle control. Medium and the treatment compounds were replaced every 48 hours. The experiment was repeated three times independently. To terminate the experiment, medium and unattached cells were removed, and the remaining cells were washed in PBS and fixed with glutaraldehyde for 15 minutes. The fixed cells were incubated with crystal violet solution (0.1% crystal violet, 20% CH_3_OH) for 1 hour. The amount of dye taken up by the monolayer, proportional to the number of viable cells attached to the well bottom, was quantified by extracting the color with 10% acetic acid and measuring the absorbance at a wavelength of 595 nm. The linear correlation between the absorbance and the number of cells was verified by performing a standard curve. Relative cell growth was determined by normalizing to the untreated controls after background (without cells) subtraction.

### Cell proliferation assay

Melanoma cells were seeded in triplicates with 500–1,000 cells per well and treated with DCA at the given concentrations and vehicle control for 96 hours. Proliferation was then measured by detecting BrdU after 12 hours of incorporation into cellular DNA. The procedure was conducted according to the protocol provided with the BrdU Cell Proliferation Assay Kit (Cell Signaling Technology®).

### Annexin V-FITC apoptosis detection

Apoptosis detection was performed using an Annexin V-FITC apoptosis detection kit (BD Bioscience), according to the provided protocol. Cells were harvested and washed twice in cold PBS. The cells were then transferred to another tube, spun down and resuspended in binding buffer. From the resuspension, 5 × 10^5^ cells were transferred to FACS tubes and stained with Annexin V-FITC and propidium iodide (PI). After 30 minutes incubation, flow cytometry was performed on a Cytomics FC 500 MPL instrument (Beckman Coulter). Unstained cells were included as control.

### Induction of *in vitro* acquired vemurafenib resistance

Acquired resistance to vemurafenib was induced in seven cultures derived from four BRAF^V600E^-mutant, vemurafenib-sensitive melanoma cell lines (ED-013, ED-071, ED-196 and SK-MEL-28). Cells were cultured in increasing concentrations of vemurafenib until they grew steadily in a concentration above the IC_50_, and were then maintained in medium containing vemurafenib.

### Pyrosequencing

Pyrosequencing of mutation hotspots in *BRAF* and *NRAS* was performed on a PyroMark Q24 platform (Qiagen), using PyroMark Gold Q24 Reagents (Qiagen). The primer sequences are listed in Additional file 1: Table S1.

### PGC1α expression analysis

Total RNA was isolated using RNeasy mini kit (Qiagen) and cDNA was synthesized with the SuperScript™ III Reverse Transcriptase kit (Invitrogen). Oligo dT24 and random hexamers were used as primers for cDNA synthesis. Gene expression of PGC1α was determined with quantitative real-time PCR on Roche LightCycler 2.0 using LigthCycler FastStart DNA Master^PLUS^ SYBR Green I kit (Roche). The primer sequences were: PPARGC1A_2241F: 5′-GCTGTACTTTTGTGGACGCA-3′ and PPARGC1A_2306R: 5′-GGAAGCAGGGTCAAAGTCAT-3′. The expression was normalized to the expression of the housekeeping gene *RPLP0*. The primer sequences were: RPLP0_433F: 5′-ACTAAAATCTCCAGGGGCACC-3′ and RPLP0_547R: 5′-ATGACCAGCCCAAAGGAGAA-3′. The two melanoma cell lines ED-050 and SK-MEL-28 were included as positive and negative controls, respectively [4].

### Statistical analysis

Differences between independent data sets were determined with Student’s t-test. One-way matched-samples ANOVA was used for statistical analysis of variance between different treatments (vehicle control, DCA, vemurafenib and the combination of DCA and vemurafenib). Tukey’s honest significance difference (HSD) multi-comparison test was used to determine statistical significance. Pearson’s correlation coefficient was used to determine correlation between DCA sensitivity and metabolic parameters. A value of 1 indicated a positive correlation, 0 no correlation, and −1 a negative correlation.

The experiments performed in this study involved only commercially available cell lines and therefore required no ethics committee approval.

## Results

### Metabolic characterization of melanoma cell lines and primary melanocytes

**Figure 1:**
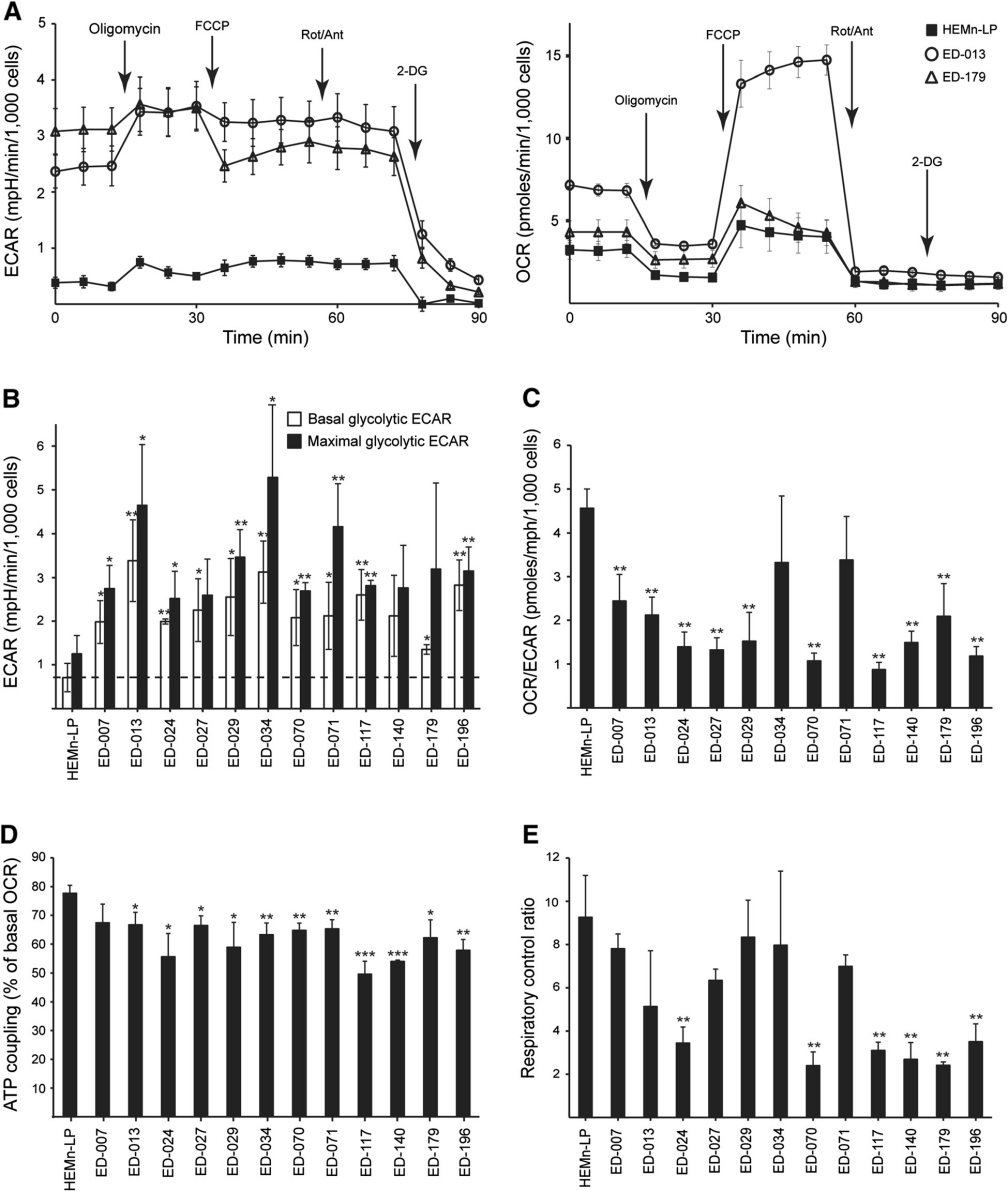
**Metabolic characterization of melanoma cell lines and primary melanocytes. A**, Metabolic profiles of two melanoma cell lines (ED-013 and ED-179) and human epidermal melanocytes (HEMn-LP), based on Seahorse XF96 measurements. Depicted are the ECAR (left panel) and the OCR (right panel) measurements during successive addition of oligomycin (1 μM), FCCP (1 μM), rotenone/antimycin (1 μM/1 μM) and 2-DG (100 mM). Data constitute 6 parallel measurements, and are representative of three independent experiments. **B**, Basal and maximal glycolytic ECAR values for melanoma cell lines and human primary melanocytes (HEMn-LP). The ECAR measured after addition of 2-DG (non-glycolytic ECAR) was subtracted from all values. The dashed line indicates the basal ECAR of HEMn-LP. **C**, Ratios between basal mitochondrial OCR and basal glycolytic ECAR. **D**, ATP coupling representing the fraction of the basal OCR used for ATP production (fraction inhibited by oligomycin). **E**, The respiratory control ratio, denoting the ratio between the maximal mitochondrial OCR and the proton leak (OCR after addition of oligomycin). (**B** – **E**), Values are the means of three independent measurements ± standard deviation. Students t-test was used to determine differences between HEMn-LP and melanoma cell lines (*p < 0.05; **p < 0.01; ***p < 0.001).

Metabolic profiling of 12 melanoma cell lines and primary human melanocytes (HEMn-LP) was performed using the Seahorse FX96 analyzer. This instrument performs real-time measurements of the extracellular acidification rate (ECAR) and the oxygen consumption rate (OCR), which are indirect measures of glycolytic activity and mitochondrial respiration, respectively [23]. The measurements were performed under basal conditions and during the successive addition of five metabolic modulators (Figure 1A). Compared with normal melanocytes, 11 out of 12 cell lines presented with higher glycolytic rates, as indicated by higher basal glycolytic ECARs, showing that the Warburg effect is a general characteristic of melanoma cells. Furthermore, nine of the cell lines exhibited higher maximal glycolytic capacities compared with melanocytes (Figure 1B). With few exceptions, there were no significant differences in basal and maximal mitochondrial respiration between melanocytes and melanoma cells (Additional file 2: Figure S2). According to the OCR-to-ECAR ratios (OCR/ECAR; Figure 1C), the relative contribution from mitochondrial respiration to ATP production was lower in 10 of the melanoma cell lines compared with melanocytes.

Mitochondrial efficiency (ATP coupling) and performance (respiratory control ratio) were estimated from measurements of two parameters for mitochondrial function; proton leak and the maximal mitochondrial OCR [24]. The proton leak was determined after the addition of the ATP synthase inhibitor oligomycin, and the maximal mitochondrial OCR was determined after the addition of the mitochondrial uncoupler FCCP. This analysis showed a significantly lower ATP coupling in melanoma cells compared with melanocytes (p < 0.05; ED-007: p = 0.07), suggesting a higher proton leak and a less efficient production of ATP relative to the level of oxygen consumption (Figure 1D). Furthermore, six of the melanoma cell lines also had significantly lower respiratory control ratios than melanocytes (p < 0.01, Figure 1E), indicative of a poor mitochondrial performance.

### DCA shifts the metabolism towards mitochondrial respiration and reduces ATP levels

**Figure 2:**
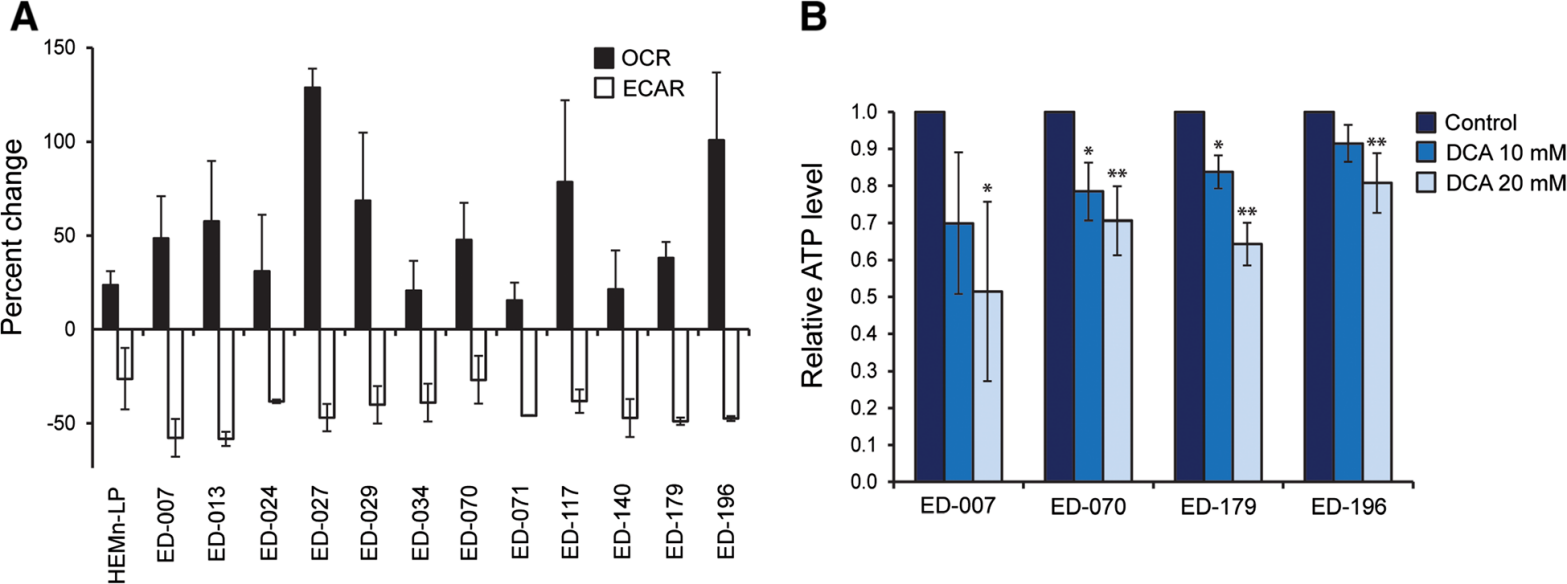
**Effects of DCA on metabolism and ATP levels. A**, Relative changes in basal mitochondrial OCR and glycolytic ECAR induced by treatment with 10 mM DCA for 2 hours. **B**, Relative ATP levels after treatment with DCA (10 or 20 mM) for 24 hours. One-way matched-samples ANOVA was used for statistical analysis and Tukey’s HSD test was used to determine statistical significance (*p < 0.05; **p < 0.01).

To determine the effect of DCA on melanoma cell metabolism, we analyzed the panel of 12 cell lines using the Seahorse XF96 analyzer. After treatment with 10 mM DCA for 2 hours, all cell lines responded with a reduction in ECAR and an increase in OCR (Figure 2A), indicating a shift towards mitochondrial respiration. The ECAR response was similar among the cell lines, whereas there was a large variation in the OCR response (Figure 2A). The relative changes in ECAR, OCR and OCR/ECAR in response to DCA were concentration dependent (Additional file 3: Figure S3).

To determine if the shift in metabolism interfered with energetic homeostasis, we measured ATP levels in melanoma cells after treatment with 0.1, 1 or 10 mM DCA for 2 hours. All three cell lines tested were able to sustain ATP levels after treatment with low concentrations of DCA (0.1-1 mM), whereas a trend towards reduced ATP was observed in cultures treated with 10 mM DCA (Additional file 3: Figure S3). When cells were treated with DCA (10 or 20 mM) for 24 hours, a significant concentration-dependent decrease in ATP was observed (Figure 2B), indicating a gradual exhaustion of the metabolic system.

### DCA reduces the growth of melanoma cells independent of genetic-driver and PGC1α-expression status

**Figure 3:**
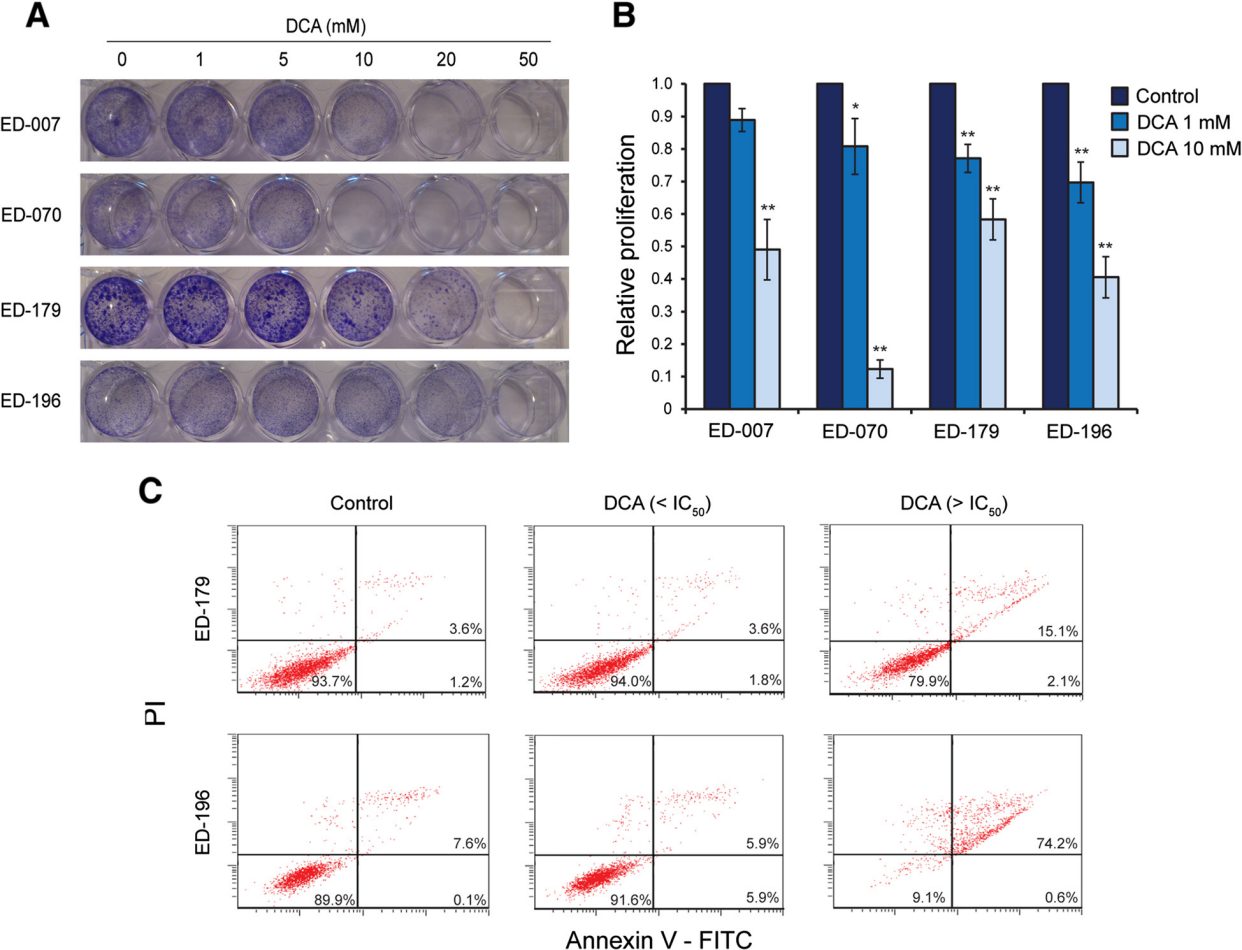
**Effects of DCA on growth**, **proliferation and apoptosis. A**, Representative selection of crystal violet stains of four melanoma cell lines (ED-007, ED-070, ED-179 and ED-196) treated with increasing concentrations of DCA (1–50 mM) for 96 hours. **B**, Proliferation determined by the incorporation of BrdU after treatment with DCA (1 and 10 mM) for 96 hours. One-way matched-samples ANOVA was used for statistical analysis and Tukey’s HSD test was used to determine statistical significance (*p < 0.05; **p < 0.01). **C**, Annexin V and PI based flow cytometric detection of apoptosis in ED-179 and ED-196 cells after treatment with DCA at concentrations below (10 mM) and above (100 mM) the IC_50_ for 96 hours.

To assess a possible clinical application of DCA for treatment of melanoma, we studied its effects on different parameters for cellular growth. The entire panel of cell lines and primary melanocytes were treated with a range of DCA concentrations (0.5-100 mM) for 96 hours (see Figure 3A for representative results). All cell lines showed a concentration-dependent reduction in growth, with IC_50_ values in the range of 9–38 mM (Table 1), compared with an IC_50_ value of 70 mM for primary melanocytes (p < 0.001). There was no correlation between the response to DCA and either the *BRAF*/*NRAS* mutation status or the expression levels of PGC1α (Table 1).

**Table 1:** **DCA IC**_**50**_ **values**, ***BRAF***/***NRAS*** **status and PGC1α expression**

**Cell line**	**DCA IC**_**50**_ **(mM)** ^**4**^	***BRAF/*** ***NRAS*** **Status** ^**1**^	**PGC1α expression** ^**2,4**^
**ED**-**070**	8.9 ± 0.6***	NRAS^Q61L^	1.0 ± 0.2
**ED**-**007**	12.2 ± 2.2***	WT^3^	0.6 ± 0.0
**ED**-**071**	12.3 ± 3.6***	BRAF^V600E^	0.0 ± 0.0
**ED**-**034**	12.7 ± 1.7***	BRAF^L597S^	1.0 ± 0.2
**ED**-**013**	14.4 ± 2.0***	BRAF^V600E^	1.9 ± 0.3
**ED**-**027**	17.7 ± 2.1***	BRAF^V600E^	0.5 ± 0.1
**SK**-**MEL**-**28**	20.0 ± 4.5***	BRAF^V600E^	0.0 ± 0.0
**ED**-**179**	20.6 ± 1.8***	NRAS^Q61R^	0.3 ± 0.0
**ED**-**024**	21.9 ± 1.6***	NRAS^Q61L^	0.0 ± 0.0
**ED**-**140**	23.9 ± 2.0***	WT	0.4 ± 0.1
**ED**-**050**	24.1 ± 3.1***	WT	2.7 ± 0.5
**ED**-**029**	29.7 ± 5.1***	BRAF^V600K^	1.3 ± 0.2
**ED**-**196**	35.8 ± 3.2***	BRAF^V600E^	1.5 ± 0.5
**ED**-**117**	37.6 ± 2.2***	BRAF^V600E^	1.2 ± 0.1
**HEMn**-**LP**	69.1 ± 6.4	WT	1
**ED**-**013**-**R1**	12.6 ± 3.0***	BRAF^V600E^	
**ED**-**013**-**R2**	13.6 ± 2.4***	BRAF^V600E^	
**ED**-**071**-**R1**	12.2 ± 0.9***	BRAF^V600E^	
**ED**-**071**-**R2**	13.8 ± 3.7***	BRAF^V600E^	
**SK**-**MEL**-**28**-**R1**	23.1 ± 4.6***	BRAF^V600E^	
**SK**-**MEL**-**28**-**R2**	26.2 ± 8.0**	BRAF^V600E^	

**p < 0.01; ***p < 0.001 when compared with HEMn-LP.

^1^BRAF/NRAS status are in accordance with published results [25].

^2^Relative to PGC1α expression in normal melanocytes.

^3^wild type.

^4^DCA IC_50_ values and PGC1α expression represent the means of three independent measurements ± standard deviation.

To further characterize the effects of DCA on cellular growth, we measured the incorporation of BrdU in cells treated with 1 or 10 mM DCA for 96 hours. As shown in Figure 3B, all four cell lines tested responded with reduced proliferation, in the range of 11-30% at 1 mM and of 42-88% at 10 mM. We also measured the apoptotic response to DCA by flow-cytometric analysis of annexin V levels. At concentrations of DCA below IC_50_, the number of annexin V-positive cells did not increase after 96 hours and up to 3 weeks. In contrast, treatment with concentrations above the IC_50_ increased the number of cells positive for both annexin V and PI, indicating induction of cell death already after 96 hours (Figure 3C).

### DCA potentiates the effect of vemurafenib on BRAF^V600E^-mutant melanoma cells

To investigate if DCA can be used to improve the efficiency of chemical BRAF inhibitors for treatment of melanoma, we tested various combinations of DCA and vemurafenib on cellular growth. Treatment of four BRAF^V600E^-mutant cell lines with vemurafenib (0.05-5 μM) for 96 hours revealed IC_50_ values from 0.5 to 4.5 μM, consistent with data from previous studies [26]. When exposing primary melanocytes to the same treatment, we did not reach the IC_50_ value for these cells, even with the highest concentration tested (5 μM), confirming the specificity of the compound.

**Figure 4:**
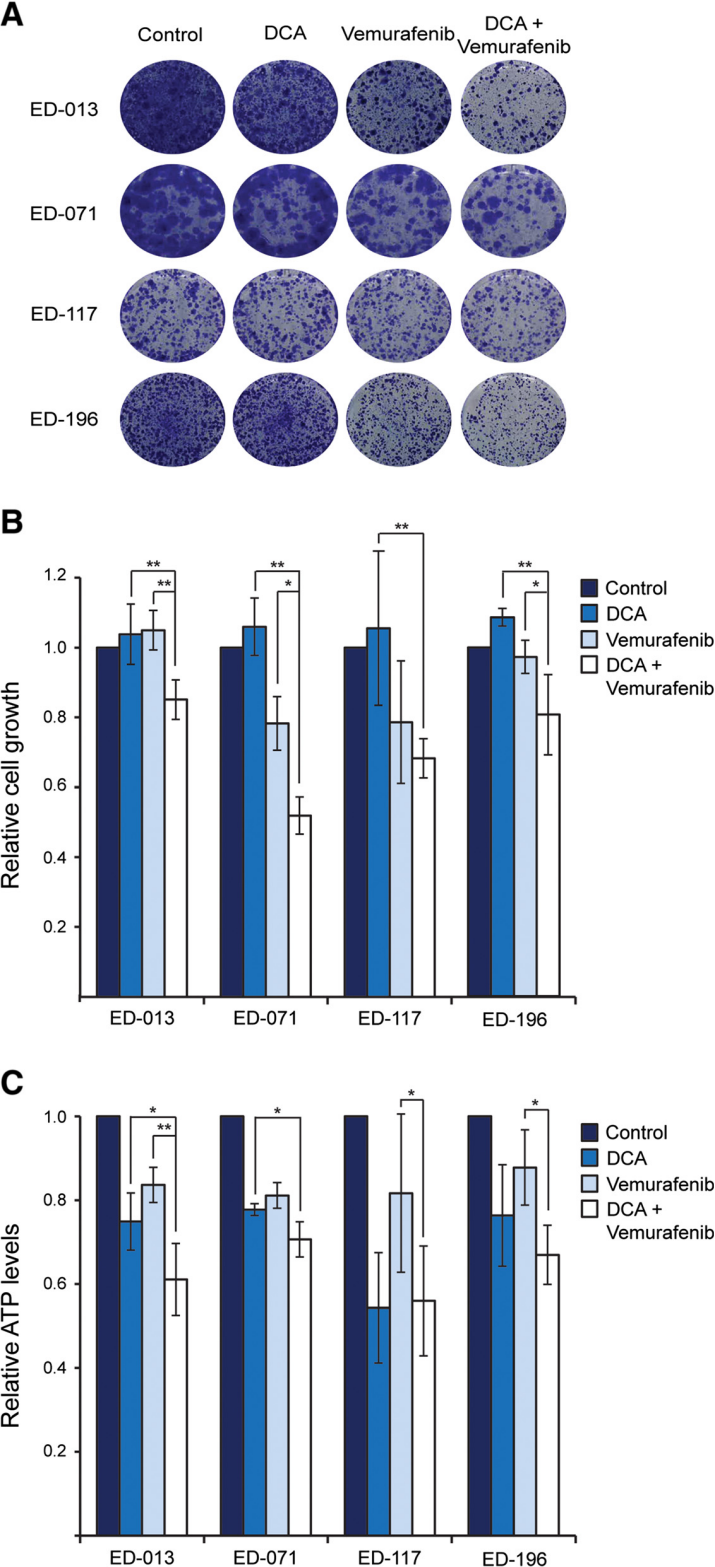
**Effects of combined treatment with DCA and vemurafenib. A**, **B**, Four melanoma cell lines (ED-013, ED-071, ED-117 and ED-196) were treated with DCA (1 mM), vemurafenib (50 nM for ED-071 and ED-117; 100 nM for ED-013 and ED-196) or the combination for 2 weeks. **A**, Crystal violet staining results representative of three independent experiments. **B**, Quantification of the data exemplified in A. **C**, Relative ATP levels in cells treated with DCA (IC_50_), vemurafenib (IC_50_) or the combination for 24 hours. **B**, **C**, Data represent means ± standard deviation of three independent measurements. One-way matched-samples ANOVA was used for statistical analysis and Tukey’s HSD test was used to determine statistical significance (*p < 0.05; **p < 0.01).

When cells were treated with 1 mM DCA in combination with low concentrations of vemurafenib (<IC_50_), the reduction in cellular growth was more pronounced than with DCA or vemurafenib alone (p < 0.05; Figure 4A, B). DCA did not potentiate the effect of vemurafenib in ED-117 cells, which may be attributed to the inherent resistance of these cells to DCA (IC_50_ 38 mM; Table 1). At IC_50_ concentrations, both DCA and vemurafenib caused a reduction in intracellular ATP levels when used as single agents and a further reduction when used in combination, although not statistically significant for all cell lines (Figure 4C).

### Vemurafenib-resistant cell lines have improved oxidative capacity and retain sensitivity to DCA

Seven cultures of vemurafenib-resistant cells were generated from four BRAF^V600E^-mutant cell lines by exposing the cells to increasing concentrations of vemurafenib. Cells were considered resistant when they could continuously be propagated at a concentration of vemurafenib above the IC_50_. DNA was isolated from all seven cultures and tested for *BRAF* copy number gain and secondary *NRAS* mutations, which are two well-described mechanisms of acquired resistance to vemurafenib [27,28]. Pyrosequencing revealed an increase in the ratio of *BRAF*^*V600E*^ to *BRAF*^*WT*^ in one of the resistant cell lines (ED-013-R2) compared with the parental cell line (Additional file 4: Figure S4). No *BRAF* or *NRAS* alterations were found in the remaining resistant cell lines.

**Figure 5:**
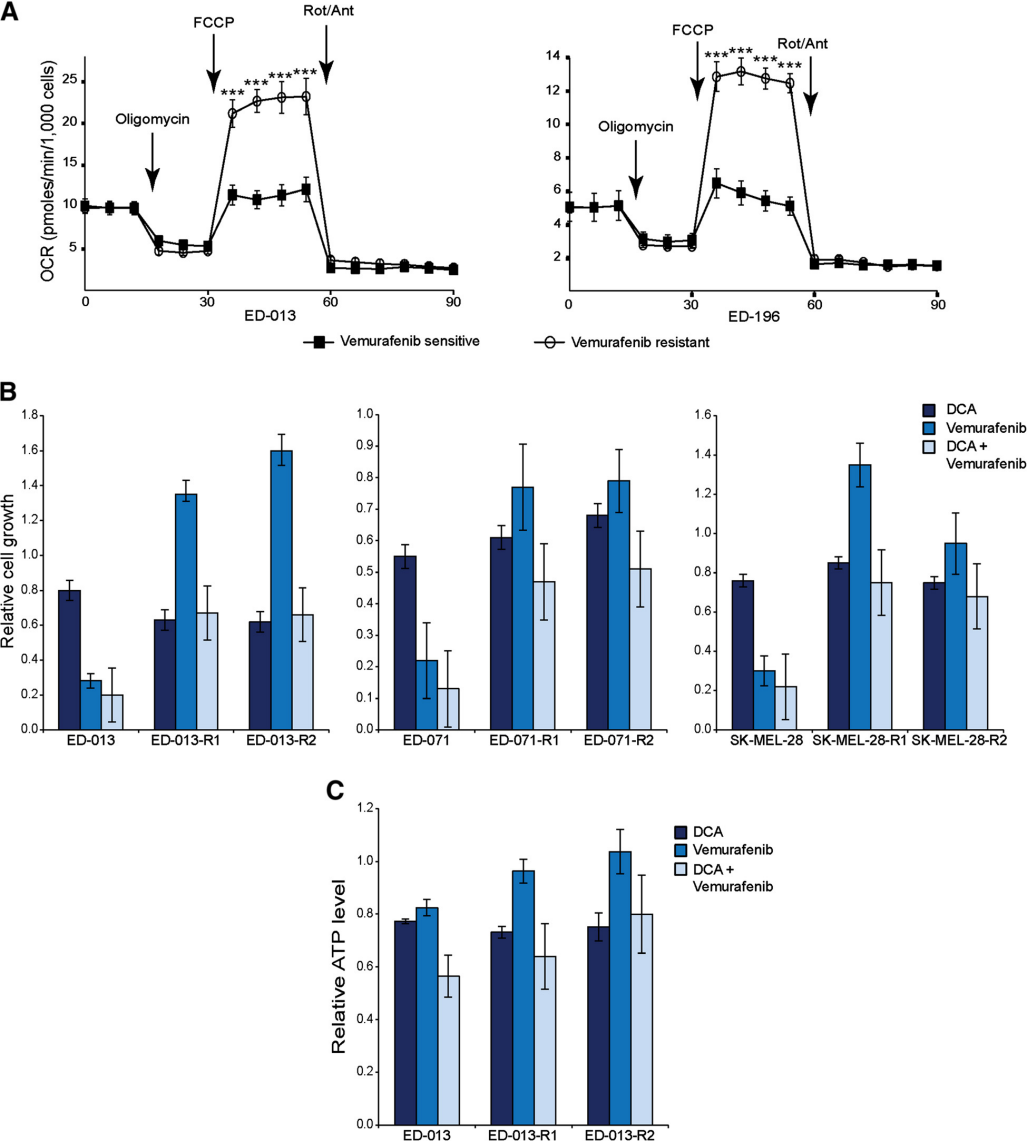
**Response of vemurafenib**-**resistant melanoma cell lines to DCA. A**, Metabolic profiles of two vemurafenib-resistant melanoma cell lines (ED-013-R1 and ED-196-R) compared with their parental cell lines (ED-013 and ED-196). The panels indicate the basal OCR and the changes during successive addition of oligomycin (1 μM), FCCP (1 μM), rotenone/antimycin (1 μM/1 μM) and 2-DG (100 mM). Data constitute 6 parallel measurements and are representative of three independent experiments. **B**, Relative cell growth of vemurafenib sensitive cell lines (ED-013, ED-071 and SK-MEL-28) and their vemurafenib resistant sub-cultures (denoted R1 and R2) after treatment with DCA (10 mM), vemurafenib (1 μM) or the combination for 96 hours. **C**, Relative ATP levels after treatment with DCA (IC_50_), vemurafenib (IC_50_) or the combination for 24 hours.

Metabolic characterization of two of the resistant cell lines (ED-013-R1 and ED-196-R) using the Seahorse XF96 analyzer showed that both resistant cell lines had a transformed metabolic profile with a significantly increased maximal respiratory capacity (Figure 5A), but no changes in basal respiratory OCR, ATP coupling or non-mitochondrial OCR. Interestingly, when tested for sensitivity to DCA, the vemurafenib-resistant cell lines all had IC_50_ values similar to those for the parental cell lines (Table 1).

Figure 5B illustrates the sensitivity of vemurafenib-resistant cell lines to DCA and vemurafenib, as single agents or in combination, compared with the parental cell lines. The growth of the resistant cell lines was slightly decreased, unaffected or even increased in the presence of vemurafenib after 96 hours, whereas the sensitivity to DCA was similar to the parental cells, both in the presence and absence of vemurafenib (Figure 5B). Similar results were obtained when measuring changes in ATP levels after treatment with the same drugs for 24 hours (Figure 5C). All together, these data show that the vemurafenib-resistant melanoma cells retained sensitivity to DCA, despite the change in their metabolic profile.

## Discussion

Metabolic targeted therapy for cancer has been primarily focused on targeting the energy supply through inhibition of glycolysis. However, the recognition that mitochondria may be active contributors to melanoma progression has increased the attention on oxidative metabolism as a potential therapeutic target [10,13,29]. DCA promotes PDK-dependent activation of PDH, reversing lactate production in favor of influx of pyruvate into the mitochondria [15,18]. Through this mechanism, DCA improves the coupling between glycolysis and mitochondrial respiration, which will have a greater impact on cells with a deficient coupling, such as cancer cells [18]. All melanoma cell lines examined in our study responded to DCA with reduced lactate production and an increased OCR. This shift towards mitochondrial respiration was expected to optimize substrate utilization and lead to a more efficient energy yield, but instead led to a significant drop in ATP levels despite an unaffected or even increased mitochondrial ATP coupling. The observed reduction in ECAR in response to DCA suggests that inhibition of glycolysis could be a major contributor to energy deprivation. A glycolysis-inhibitory mechanism of DCA has not been previously described. However, it has been demonstrated that pyruvate kinase, the last ATP-producing site in the glycolytic pathway, is negatively regulated by acetyl coenzyme A (acetyl-CoA) [30]. As PDH activation directly increases the formation of acetyl-CoA [31], this could explain the DCA-mediated inhibition of glycolysis. The structural similarity between DCA and pyruvate [32] could also imply a direct inhibition of glycolysis by DCA, possibly through an allosteric feedback mechanism.

The metabolic response to DCA was accompanied by reduced proliferation of melanoma cells, independent of the genetic driver status and metabolic profiles of these cells. Several previous studies have demonstrated an apoptotic effect of DCA on cancer cells [13,18,19,32–34]. However, in accordance with our results, the apoptotic response was only triggered at concentrations too high to be clinically relevant [32]. To further explore the clinical relevance of DCA to melanoma treatment, we examined the efficacy of this agent in combination with the BRAF inhibitor vemurafenib. These experiments demonstrated a potentiating effect of DCA on the growth inhibition of BRAF^V600E^-mutant melanoma cells. At low concentrations of DCA that alone had no effect on cell growth, the combination with low concentrations of vemurafenib had a significantly stronger growth-reducing effect than vemurafenib alone. This potentiating effect of DCA was also reflected in the reduction of ATP levels. Biochemical analysis has demonstrated the ability of BRAF^V600E^ to uncouple the LKB1-AMPK energy sensing pathway, promoting resistance to energy deprivation and preventing an apoptotic response [8,9]. Treatment with BRAF inhibitors restores this pathway [35] and may, therefore, potentiate the response to compounds that reduce the generation of ATP. Both DCA and vemurafenib suppress glycolytic activity in melanoma cells and thus render them more dependent on mitochondrial respiration [6]. As glycolysis accounts for a large fraction of the total energy production in these cells, inhibition of this process will place a high demand on the oxidative system for ATP production. The lower performance of the mitochondria in melanoma cells could explain the inability of these cells to sustain ATP levels in the presence of DCA and vemurafenib. The cooperative effect of these compounds in lowering ATP levels suggests that the energetic threshold promoting growth arrest or cell death in melanoma cells can be reached with lower concentrations of vemurafenib in the presence of DCA.

Previous studies have investigated the ability of metabolic modulators to improve the therapeutic effect of BRAF inhibitors for treatment of melanoma. The combination of PLX4720 (a vemurafenib analogue) with either of the two anti-diabetic biguanides, metformin and phenformin, showed synergistic inhibition of melanoma cell viability [35,36]. Both agents impair ATP synthesis through inhibition of the mitochondrial complex I activity, leading to a reduction in the ATP to ADP ratio and activation of the LKB1-AMPK pathway to suppress growth [35,36]. Unlike DCA, metformin and phenformin both stimulate glycolysis and lactic acid production [37,38], which could explain the growth-stimulating effects of metformin on some melanoma cell lines when used as a single agent. In addition, the concentrations at which metformin was effective were above a therapeutically relevant level [35]. Phenformin was significantly more potent than metformin [36], but has been associated with a high risk of lactic acidosis [39], and was taken off the market for treatment of type 2 diabetes in many countries. DCA, on the other hand, was here demonstrated to potentiate the effect of vemurafenib at concentrations down to 1 mM, and was previously shown to have few adverse effects when administered to patients [17,19,40]. These findings allude to a therapeutic potential of DCA as a co-drug for vemurafenib treatment of BRAF^V600E^-mutant melanoma. This was reinforced by the demonstration that sensitivity to DCA was retained in melanoma cell lines with acquired resistance to vemurafenib. Although resistant cells showed an altered metabolic profile with significantly increased maximal mitochondrial respiration, as also shown by Corazao-Rozas *et al*. [41], they were as sensitive to DCA as the parental vemurafenib-sensitive cells. Therefore, DCA could possibly provide a strategy to prevent the appearance of vemurafenib-tolerant subpopulations during initial treatment and thereby postpone or prevent the development of resistance.

## Conclusions

We here provide a more elaborate understanding of the effects of DCA on the metabolism and growth of melanoma cells. The ability of DCA to lower ATP levels and melanoma growth appears to potentiate the effect of vemurafenib, a drug already used in the clinic for treatment of BRAF^V600E^-mutant metastatic melanomas. Importantly, melanoma cells with acquired resistance to vemurafenib retained their sensitivity to DCA. These findings should encourage further investigation of this drug combination and the *in vivo* application of DCA.

## Additional files

Additional file 1: Table S1. Pyrosequencing primers for amplification and sequencing of *BRAF* and *NRAS* mutation hotspots. Forward, reverse and sequencing primers are denoted F, R and S, respectively. Additional file 2: Figure S2. Basal and maximal mitochondrial OCR values for melanoma cell lines and human epidermal melanocytes (HEMn-LP). The OCR measured after addition of rotenone/antimycin A (non-mitochondrial OCR) was subtracted from all values. The dashed line indicates the basal OCR of HEMn-LP. The indicated values are means of three independent measurements ± standard deviation. Students t-test was used to determine differences between HEMn-LP and the melanoma cell lines (*p < 0.05; **p < 0.01; ***p < 0.001). The maximal mitochondrial OCR for ED-034 was not indicated due to a very large variation among four independent experiments, ranging from 6.19 to 58.36 pmoles/min/1,000 cells. Additional file 3: Figure S3. Metabolic response of melanoma cells to DCA Relative response in ECAR, OCR, OCR/ECAR and ATP levels after treatment with DCA (0.1, 1 and 10 mM) for 2 h. The error bars in the first three panels represent standard deviations of three repeated measurements of six parallel samples. The error bars in the lower panel represent the standard deviation of three independent experiments. One-way matched-samples ANOVA was used for statistical analysis and Tukey’s HSD test was used to determine statistical significance (*p < 0.05; **p < 0.01). Additional file 4: Figure S4. Allele status of *BRAF*. Pyrosequencing of the *BRAF* c.1799 T > A mutation site in ED-013 and the vemurafenib resistant derivative ED-013-R2. The increased *BRAF* ^*V600E*^-to-*BRAF* ^*WT*^ ratio in ED-013-R2 indicates a copy number gain, which could explain the resistance to vemurafenib.

## Abbreviations

(Acetyl-CoA): Acetyl coenzyme A
(AMPK): AMP activated protein kinase
(DCA): Dichloroacetate
(ECAR): Extracellular acidification rate
(HEMn-LP): Human epidermal melanocytes
(IC_50_): half maximal inhibitory concentration
(LKB1): Liver kinase B1
(MITF): Microphthalmia-associated transcription factor
(OCR): Oxygen consumption rate
(PDH): Pyruvate dehydrogenase
(PDK): Pyruvate dehydrogenase kinase
