# Supplementary material for: Bioenergetic modulation with dichloroacetate reduces the growth of melanoma cells and potentiates their response to BRAFV600E inhibition
Source: J Transl Med. 2014 Sep 3;12:247. doi: 10.1186/s12967-014-0247-5 (PMC4156963; doi:10.1186/s12967-014-0247-5)
Supplement: Additional file 1: Table S1. — Pyrosequencing primers for amplification and sequencing of BRAF and NRAS mutation hotspots. Forward, reverse and sequencing primers are denoted F, R and S, respectively. [file 12967_2014_247_MOESM1_ESM.doc]

**Supplementary Table S1**

| **Target site** | **Primer name** | **Primer sequence (5’-3’)** |
| --- | --- | --- |
| *BRAF* V600E | BRAF-F1 | [Btn]-TTCATGAAGACCTCACAGTAAAAA |
|  | BRAF-R1 | GGCCAAAAATTTAATCAGTGGAA |
|  | BRAF-S1 | CCACTCCATCGAGATTT |
| *NRAS* Q61K,L,R | NRAS-F1 | [Btn]-ACCCCCAGGATTCTTACAGAAA |
|  | NRAS-R1 | CGCAAATGACTTGCTATTATTGA |
|  | NRAS-S1 | TCATGGCACTGTACTCTT |
