# Supplementary figures and images for: Bioenergetic modulation with dichloroacetate reduces the growth of melanoma cells and potentiates their response to BRAFV600E inhibition
Source: J Transl Med. 2014 Sep 3;12:247. doi: 10.1186/s12967-014-0247-5 (PMC4156963; doi:10.1186/s12967-014-0247-5)

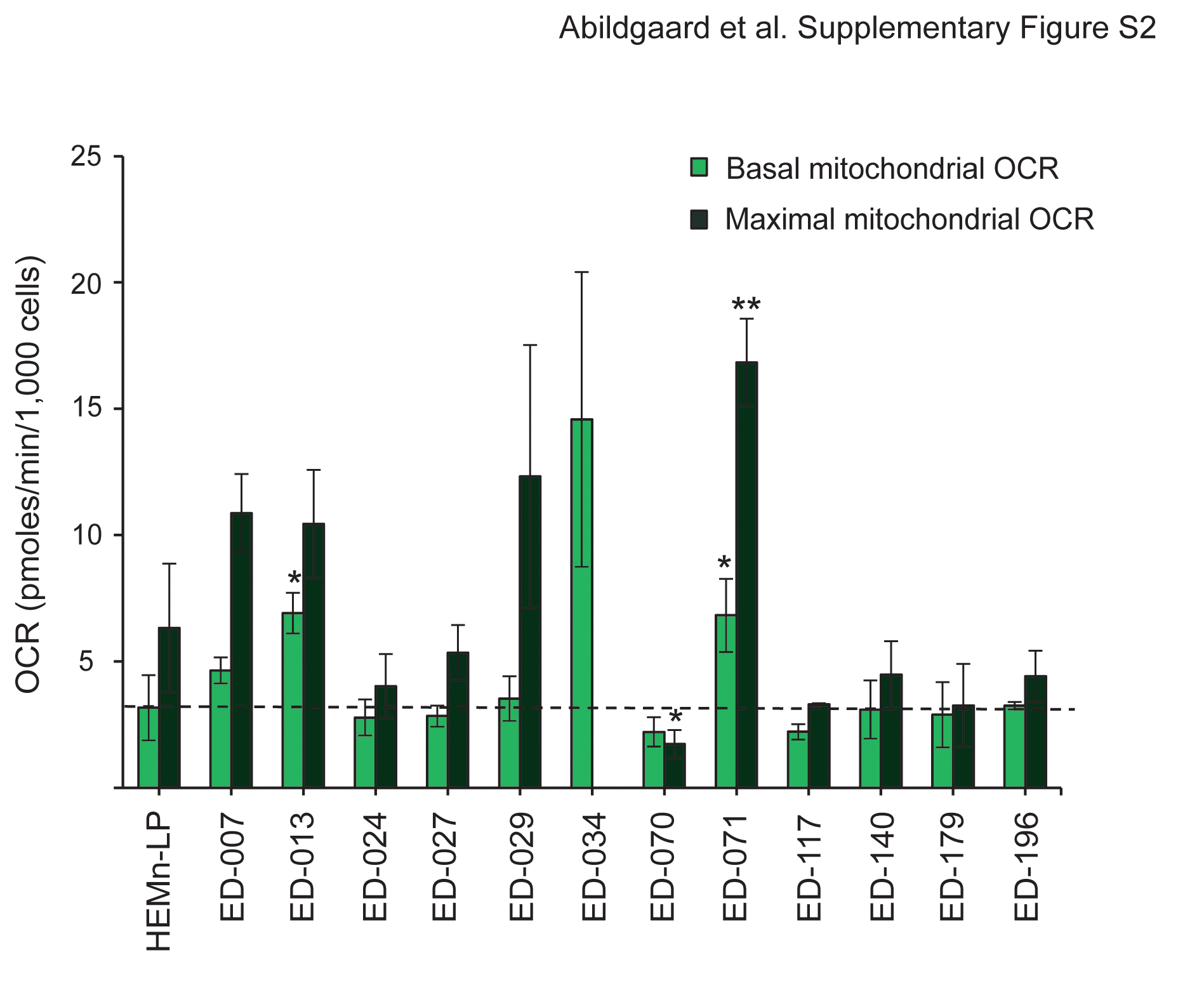

Supplement: Additional file 2: Figure S2. — Basal and maximal mitochondrial OCR values for melanoma cell lines and human epidermal melanocytes (HEMn-LP). The OCR measured after addition of rotenone/antimycin A (non-mitochondrial OCR) was subtracted from all values. The dashed line indicates the basal OCR of HEMn-LP. The indicated values are means of three independent measurements ± standard deviation. Students t-test was used to determine differences between HEMn-LP and the melanoma cell lines (*p < 0.05; **p < 0.01; ***p < 0.001). The maximal mitochondrial OCR for ED-034 was not indicated due to a very large variation among four independent experiments, ranging from 6.19 to 58.36 pmoles/min/1,000 cells. [file 12967_2014_247_MOESM2_ESM.tiff]

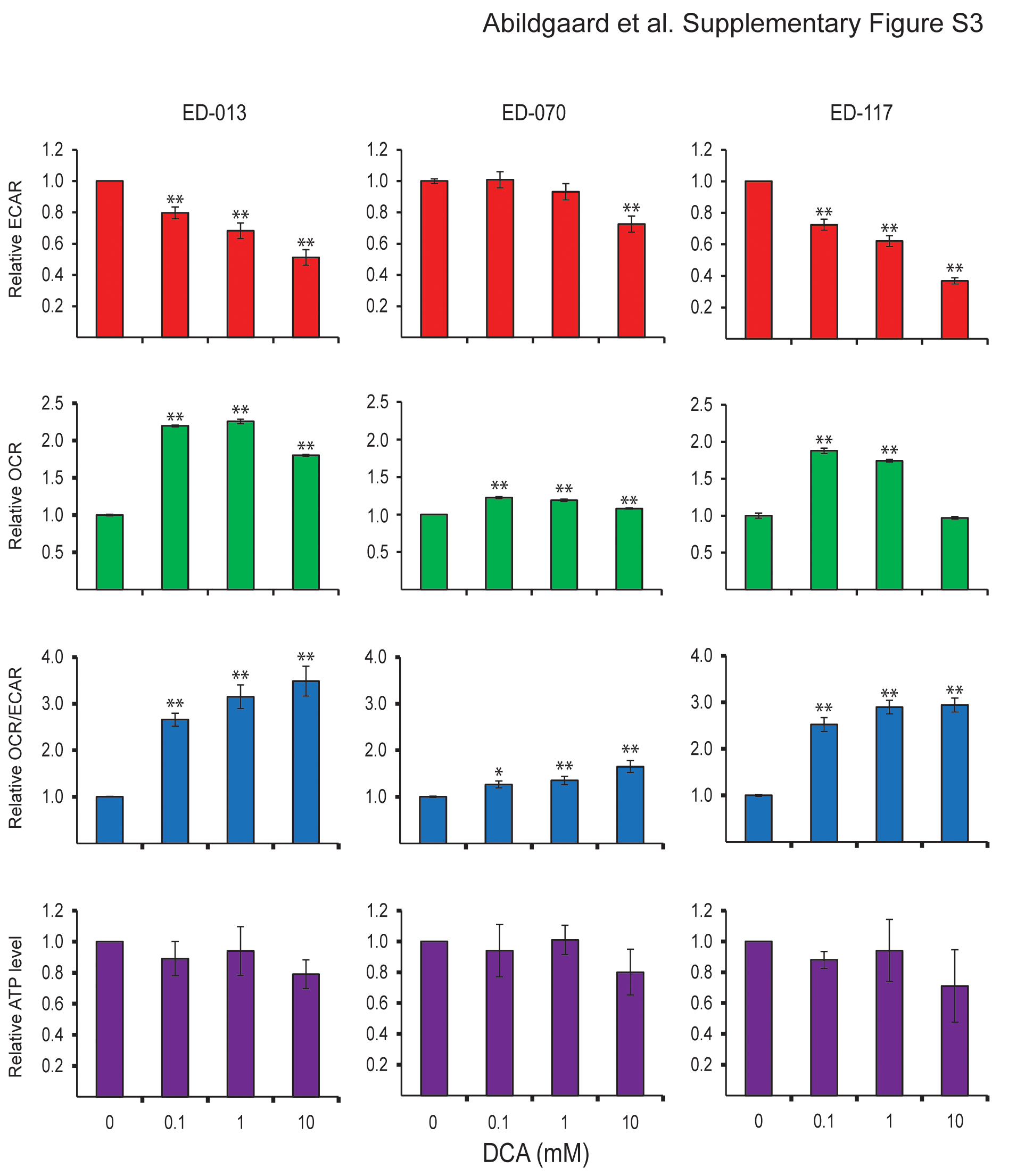

Supplement: Additional file 3: Figure S3. — Metabolic response of melanoma cells to DCA Relative response in ECAR, OCR, OCR/ECAR and ATP levels after treatment with DCA (0.1, 1 and 10 mM) for 2 h. The error bars in the first three panels represent standard deviations of three repeated measurements of six parallel samples. The error bars in the lower panel represent the standard deviation of three independent experiments. One-way matched-samples ANOVA was used for statistical analysis and Tukey’s HSD test was used to determine statistical significance (*p < 0.05; **p < 0.01). [file 12967_2014_247_MOESM3_ESM.tiff]

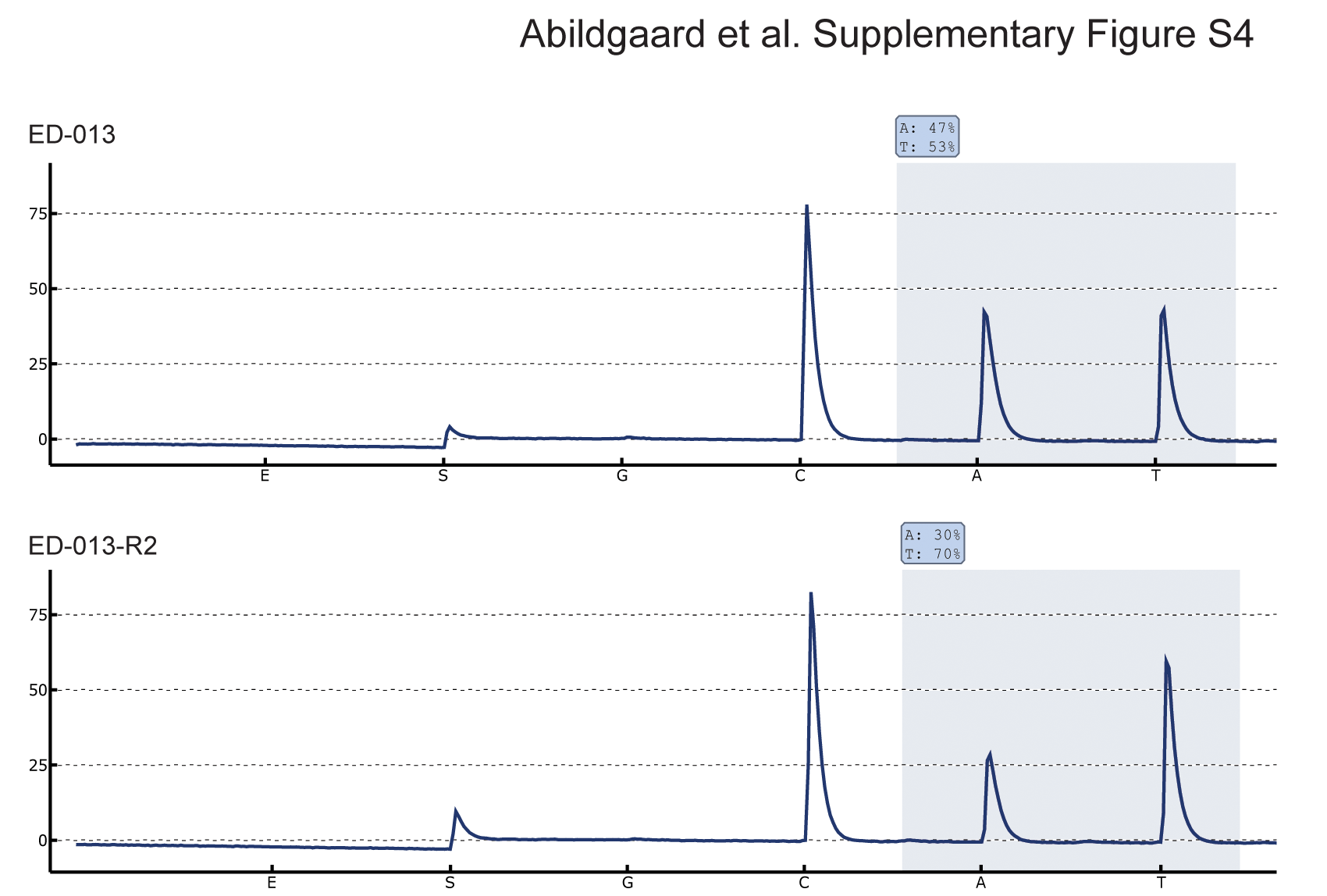

Supplement: Additional file 4: Figure S4. — Allele status of BRAF. Pyrosequencing of the BRAF c.1799 T > A mutation site in ED-013 and the vemurafenib resistant derivative ED-013-R2. The increased BRAF V600E-to-BRAF WT ratio in ED-013-R2 indicates a copy number gain, which could explain the resistance to vemurafenib. [file 12967_2014_247_MOESM4_ESM.tiff]
